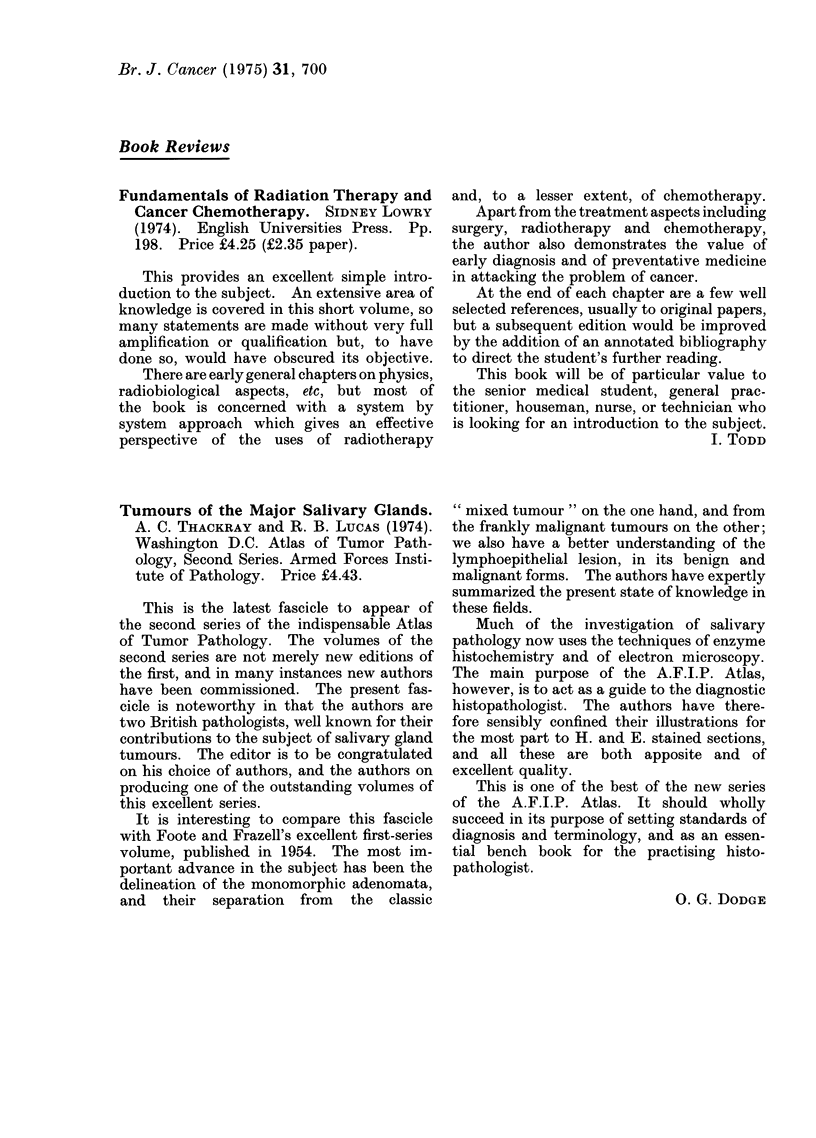# Fundamentals of Radiation Therapy and Cancer Chemotherapy

**Published:** 1975-06

**Authors:** I. Todd


*Br. J. Cancer* (1975) **31**, 700

### *Book Reviews*

**Fundamentals of Radiation Therapy and Cancer Chemotherapy.** SIDNEY LOWRY (1974). English Universities Press. Pp. 198. Price £4.25 (£2.35 paper).

This provides an excellent simple introduction to the subject. An extensive area of knowledge is covered in this short volume, so many statements are made without very full amplification or qualification but, to have done so, would have obscured its objective.

There are early general chapters on physics, radiobiological aspects, *etc*, but most of the book is concerned with a system by system approach which gives an effective perspective of the uses of radiotherapy and, to a lesser extent, of chemotherapy.

Apart from the treatment aspects including surgery, radiotherapy and chemotherapy, the author also demonstrates the value of early diagnosis and of preventative medicine in attacking the problem of cancer.

At the end of each chapter are a few well selected references, usually to original papers, but a subsequent edition would be improved by the addition of an annotated bibliography to direct the student's further reading.

This book will be of particular value to the senior medical student, general practitioner, houseman, nurse, or technician who is looking for an introduction to the subject.

I. TODD